# Utility of p63 and PTEN staining in distinguishing cervical microglandular hyperplasia from endometrial endometrioid carcinoma with microglandular/mucinous features

**DOI:** 10.1111/his.14655

**Published:** 2022-05-05

**Authors:** Batoul A. Aoun, Stephanie L. Skala

**Affiliations:** ^1^ Department of Pathology University of Michigan Ann Arbor MI USA; ^2^ Rogel Cancer Center University of Michigan Ann Arbor MI USA

**Keywords:** endometrioid carcinoma, microglandular hyperplasia, microglandular hyperplasia‐like carcinoma

## Abstract

**Aims:**

Distinction between well‐differentiated endometrial carcinoma (EMCA) with microglandular/mucinous features and benign endocervical microglandular hyperplasia (MGH) can be a diagnostic challenge, especially when tissue is limited. The immunostains used to distinguish endocervical and endometrial carcinoma are less useful when the differential diagnosis is MGH. Here, we investigate the utility of p63 and phosphatase and tensin homologue (PTEN) to aid accurate classification.

**Methods and results:**

Cases obtained from our pathology archives included 25 EMCA with mucinous/microglandular features, 26 MGH and nine atypical microglandular proliferations. Cases were assessed for glandular architecture, presence of mucinous and/or eosinophilic luminal secretions, subnuclear vacuoles, foamy histiocytes, inflammation, squamous metaplasia, cytological atypia and mitotic activity. The presence and pattern of immunohistochemical staining for p63 and PTEN was recorded. Microglandular proliferations with cytological atypia, mitotic activity, foamy histiocytes and complex glandular architecture were more commonly seen in EMCA, while small glands, bland nuclei and subnuclear vacuoles were enriched in MGH. All MGH cases displayed p63‐positive subcolumnar reserve cells and retained PTEN expression. Four EMCA cases showed non‐specific focal p63 staining either at the surface of the tumour or in areas of squamous differentiation. p63 and PTEN immunostains accurately predicted the final diagnosis for 3 atypical microglandular proliferation cases with follow‐up.

**Conclusions:**

While there are morphological characteristics that differentiate EMCA and MGH, there is frequent overlap between these entities. Nonetheless, the pattern and extent of p63 and PTEN can aid accurate classification. Consistent p63‐positive subcolumnar reserve cells were seen only in MGH.

## Graphical abstract

Based on a study of 25 endometrial endometrioid carcinomas (EMCA, A) with microglandular and/or mucinous features and 26 microglandular hyperplasia (MGH, C) samples, the pattern of p63 immunohistochemical staining is often useful for distinction between these entities. EMCA was typically negative for p63 (B). Subcolumnar p63 staining of cervical reserve cells was seen in all MGH cases (D), while focal nonspecific p63 staining was seen in 4 EMCA cases.

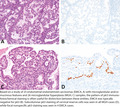

## Introduction

Distinction of benign endocervical microglandular hyperplasia (MGH) from low‐grade endometrioid carcinoma (EMCA) can constitute a diagnostic challenge, particularly when limited tissue is available for evaluation (i.e. biopsies or curettings). Microscopically, MGH is characterised by the presence of tightly packed small‐ to medium‐sized glands with minimal intervening stroma, and variable intraluminal mucin and acute inflammation. The nuclei are typically uniform with rare to absent mitotic activity [< 1 mitotic figure per 10 high‐power fields (HPF)].^1^ While MGH is not uncommon in reproductive‐age individuals, it is an unexpected finding in a postmenopausal patient in the absence of hormone therapy. Because MGH is so much less common in postmenopausal patients, care must be taken to exclude the alternative diagnosis of low‐grade EMCA, which may show microglandular architecture with bland nuclei and low mitotic activity.^2^, ^3^, ^4^, ^5^, ^6^

To distinguish between endocervical adenocarcinoma and EMCA, pathologists commonly use a battery of stains including vimentin, p16, oestrogen receptor (ER) and progesterone receptor (PR). While negative in human papillomavirus (HPV)‐associated endocervical adenocarcinoma, vimentin is positive in the majority of EMCAs.^7^, ^8^ p16 is used as a surrogate marker of high‐risk HPV infection;^9^ therefore, it is not useful in differentiating between benign endocervical proliferations and EMCA. The majority (> 80%) of low‐grade EMCAs express ER and PR,^8^ while HPV‐associated endocervical adenocarcinomas are typically negative (or only focally weakly positive) for ER and PR.^10^, ^11^ While ‘increased’ Ki‐67 proliferative activity may be expected in carcinoma compared to hyperplasia, no definitive diagnostic cut‐offs exist. Studies have shown marked overlap in the Ki‐67 proliferative activity for MGH and EMCA.^12^, ^13^ Ki67 assessment is problematic, as it is difficult to determine the nature of the immunoreactive cells.^13^ Inflammatory cells as well as actively proliferating reserve cells can have positive Ki67 staining, which may result in overestimation of the Ki67 proliferative index.

Although it may be difficult to appreciate their presence on haematoxylin and eosin (H&E)‐stained slides, subcolumnar reserve cells can be expected in MGH but not in EMCA. Cervical reserve cells are small undifferentiated pluripotential cells most conspicuous under the columnar epithelial layer of the endocervix proximal to the squamocolumnar junction. During reproductive years, these cells expand in number and undergo squamous or glandular differentiation.^14^ Reserve cells are positive for p63, and their location leads to a characteristic linear subcolumnar pattern by immunohistochemistry, while adenocarcinomas are typically negative for this marker.^15^

*PTEN* is often altered in EMCA;^16^ the absence of phosphatase and tensin homologue (PTEN) immunohistochemical expression has a sensitivity of ~75% and specificity of ~85% for predicting loss of function *PTEN* mutation.^17^ According to the Human Protein Atlas, normal endocervical and endometrial glandular cells show expression of PTEN protein (low level).^18^ To our knowledge, loss of PTEN expression has not been reported in MGH.

Herein, we describe the morphological features and p63 and PTEN immunohistochemical staining patterns in a cohort of EMCA, MGH and atypical microglandular proliferations. We aim to determine whether p63 and PTEN stains are useful to guide appropriate diagnosis of these lesions.

## Materials and methods

This study was approved by the Institutional Review Board at the University of Michigan with a waiver of informed consent due to use of archived slides and formalin‐fixed, paraffin‐embedded tissue blocks.

Sixty cases with collection dates between 2015 and 2022 were identified from our surgical pathology and consultative archives. The final cohort included 26 cases of endocervical MGH, 25 cases of EMCA with mucinous and/or microglandular features and nine cases diagnosed as ‘atypical microglandular proliferation’. Three ‘atypical microglandular proliferation’ cases had subsequent hysterectomy specimens available for review (bringing the total number of specimens examined in this study to 63). The procedures performed included endocervical curettage (*n* = 16), total hysterectomy (*n* = 19, including the three follow‐up specimens), endometrial curettage (*n* = 8), endometrial biopsy (*n* = 12), cervical biopsy (*n* = 7) and loop electrosurgical excision procedure (LEEP, *n* = 1). The distribution of procedures by diagnosis is presented in Supporting information, Table S1. Pathology reports and formalin‐fixed paraffin‐embedded tissue were available for all patients. Sections from each case were cut at 5 μm and stained with H&E. In all cases, immunohistochemical staining was performed using commercially available antibodies for p63 (clone 4A4; Ventana Medical Systems, Oro Valley, AZ, USA) and PTEN (D4.3 XP; Cell Signalling Technology, Danvers, MA, USA) with appropriate positive and negative tissue controls.

All H&E‐stained slides were reviewed for the following morphological features: (1) glandular architecture, (2) presence of mucinous and/or eosinophilic luminal secretions, (3) subnuclear vacuoles, (4) foamy histiocytes, (5) degree of inflammation, (6) luminal squamous metaplasia, (7) cytological atypia and (8) mitotic activity. Glandular architecture was classified into small, medium and large/complex glands. Cytological atypia included mild nuclear atypia [increased nuclear to cytoplasmic ratio (N:C) and inconspicuous nucleoli] and moderate nuclear atypia (nuclear pleomorphism, hyperchromasia and prominent nucleoli). Mitotic activity was assessed by counting mitotic figures in 10 consecutive high‐power fields (400× magnification; 0.16 mm^2^ per HPF). All cases were assessed for the presence and pattern/distribution of nuclear p63 staining, as well as the presence of nuclear PTEN expression (uniformly intact or loss of nuclear expression). A brief clinical history including age, menopause status, oral contraceptive use and follow‐up information was obtained from electronic medical records.

## Results

Patients’ ages in this cohort ranged from 25–93 years; 32 (55%) were postmenopausal, 24 (39%) reproductive age and four (7%) perimenopausal. The mean age for EMCA patients was 67 years, compared to 44 years for MGH.

**Figure 1:**
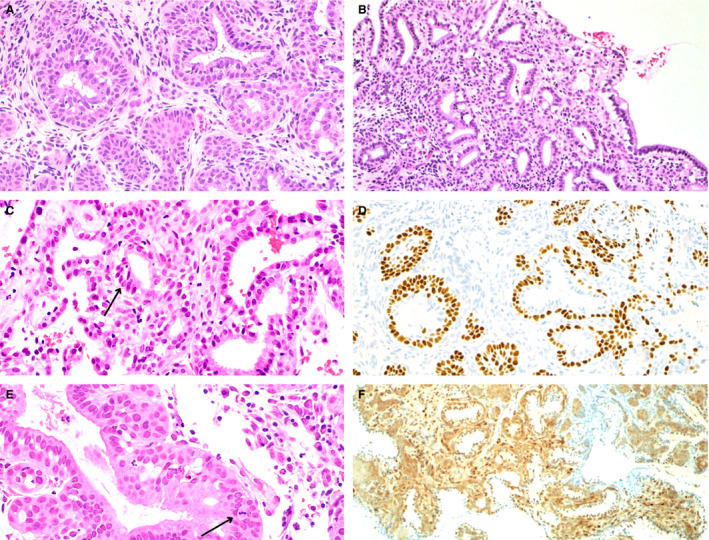
Morphological features and p63 and phosphatase and tensin homologue (PTEN) expression in microglandular hyperplasia. Endocervical microglandular hyperplasia may show a prominent reserve cell layer [**A**, haematoxylin and eosin (H&E)], although reserve cells are not always conspicuous on H&E‐stained sections. Tightly packed small to medium glands lined by epithelium with subnuclear vacuoles (**B**, H&E; **C**, H&E, black arrow) are characteristic features. Linear nuclear immunoreactivity for p63 was seen in subcolumnar reserve cells [**D**, p63 immunohistochemistry (IHC)]. Mitotic figures were rarely seen in microglandular hyperplasia (MGH) cases (**E**, H&E, black arrow). Immunohistochemical expression of PTEN was retained in MGH (**F**, PTEN IHC).

**Figure 2:**
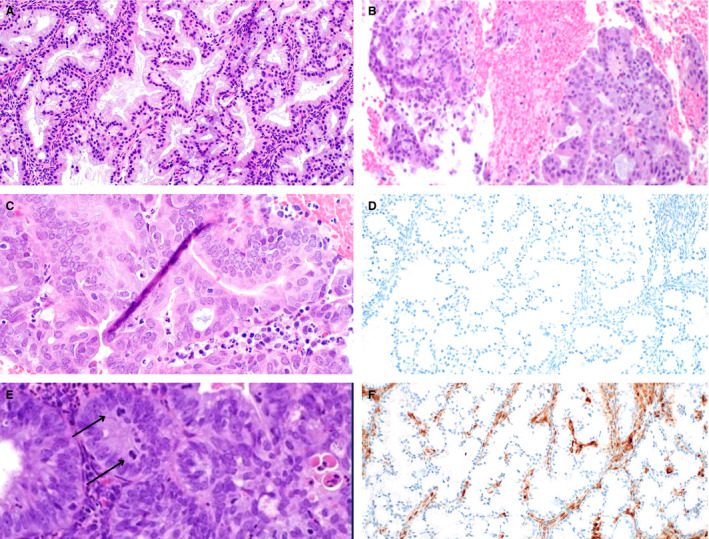
Morphological features and p63 and phosphatase and tensin homologue (PTEN) expression in endometrial carcinoma. Low‐grade (FIGO grade 1) endometrial endometrioid carcinoma sometimes shows prominent mucinous and/or microglandular features [**A**,**B**,**C**, haematoxylin and eosin (H&E)]. In contrast to microglandular hyperplasia, endometrioid carcinoma typically lacked expression of p63 [**D**, p63 immunohistochemistry (IHC)]. Mitotic figures, while more often seen in endometrioid carcinoma (**E**, H&E), could also be seen in microglandular hyperplasia (MGH). Most cases of endometrioid carcinoma showed loss of PTEN expression by immunohistochemistry (**F**, PTEN IHC).

The majority (25 of 26, 96%) of MGH lesions showed small tightly packed glands (Figure 1A–C), with only one case (4%) demonstrating medium to large tightly packed glands. Luminal secretions or mucin were seen in more than half the cases examined (15 of 26, 58%). Subnuclear vacuoles were commonly encountered in the cases examined (10 of 26, 38%). Foamy histiocytes were only present in one case (4%). Associated inflammation was present in all cases. Squamous metaplasia was seen in a subset of cases (five of 26, 19%). Cytological atypia was not appreciated. All cases of MGH demonstrated p63‐positive reserve cells (Figure 1D). Rare mitotic Figures (1–2 per 10 HPF) were seen in two MGH cases (Figure 1E). PTEN expression was intact by immunohistochemistry (Figure 1F) in all but one case.

**Figure 3:**
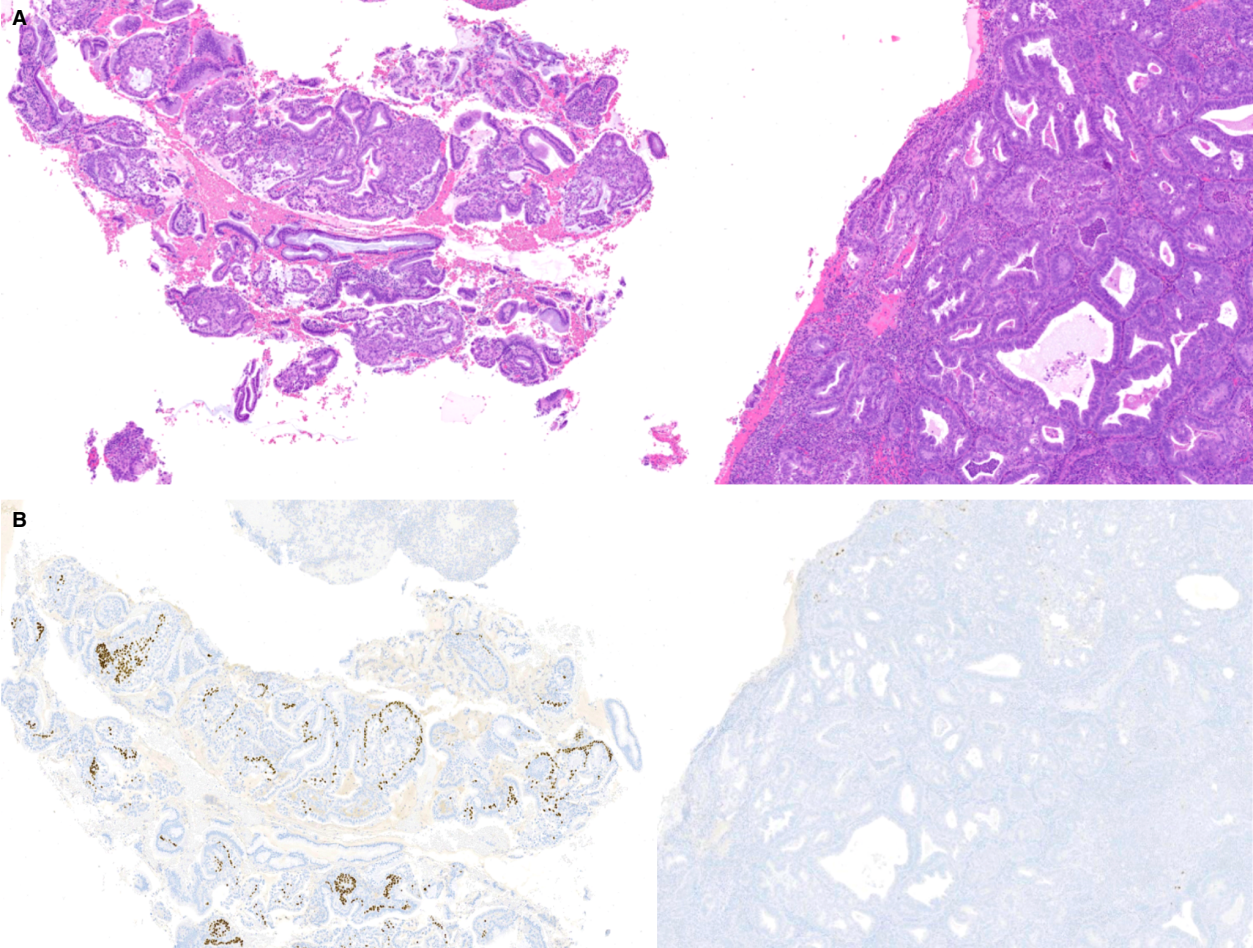
Endometrial curettings with both microglandular hyperplasia and endometrioid carcinoma. In some cases, endometrial curettings included foci of microglandular hyperplasia (MGH) (left) in addition to foci of low‐grade endometrioid carcinoma with mucinous features [right; **A**, haematoxylin and eosin (H&E)]. On low‐power examination, these two entities had similar appearances. A p63 immunostain highlighted the linear expression of endocervical reserve cells in MGH, which were not seen in areas of endometrioid carcinoma [**B**, p63 immunohistochemistry (IHC)].

In contrast, the majority of EMCA showed medium to large tightly packed glands (16 of 25, 64%) rather than small tightly packed glands (nine of 25, 36%). Common histological findings included luminal secretions or mucin (18 of 25, 72%), inflammation (22 of 25, 88%) and mild or moderate cytological atypia (23 of 25, 92%). Foamy histiocytes were seen in six cases (six of 25, 24%), and only one case demonstrated focal subnuclear vacuoles (4%). Squamous differentiation was seen in a subset of cases (five of 25, 20%). Mitotic activity of varying degrees was seen in most EMCA cases. While four cases showed focal areas of p63 positivity, the staining was present in areas of squamous differentiation (1) or a non‐specific pattern (3). No EMCA cases showed areas of subcolumnar p63 positivity as was seen in the MGH lesions. The majority of EMCA (20 of 22, 91%) demonstrated loss of PTEN expression by immunohistochemistry (IHC). Figure 2 illustrates these morphological and immunohistochemical findings. Features of MGH and EMCA are compared in Table 1. Small tightly packed glands, subnuclear vacuoles and p63 positivity were statistically significantly associated with a diagnosis of MGH, while medium to large tightly packed glands, foamy histiocytes, cytological atypia and loss of PTEN expression were statistically significantly associated with a diagnosis of EMCA. Of note, some samples showed coexisting MGH and EMCA. Figure 3 highlights the morphological similarities between these entities (Figure 3A), as well as the difference in p63 staining pattern (Figure 3B).

**Table 1:** Morphological and immunohistochemical features of microglandular hyperplasia (MGH) and endometrioid carcinoma (EMCA)

	Gland architecture		Luminal secretions/mucin	Subnuclear vacuoles	Foamy histiocytes	Inflammation	Squamous metaplasia	Cytological atypia	Mitotic figures per 10 HPF, mean (range)	p63 positivity	Loss of PTEN expression
	Small tightly packed	Medium to large tightly packed									
MGH (*n* = 26)	**25 (96%)**	1 (4%)	15 (58%)	**10 (38%)**	1 (4%)	26 (100%)	5 (19%)	0 (0%)	0.1 (0–2)	**26 (100%)**	1 (4%)*
EMCA (*n* = 25)	9 (36%)	**16 (64%)**	18 (72%)	1** (4%)	**6 (24%)**	22 (88%)	5 (20%)	**23 (92%)**	23 (1–25)	4 (16%)***	**20 (91%)** ****

Bold type indicates statistically significant differences (*P*‐values as follows: gland architecture 0.000, subnuclear vacuoles 0.003, foamy histiocytes 0.037, cytological atypia 0.000, p63 positivity 0.000, loss of phosphatase and tensin homologue (PTEN) expression 0.000) HPF, high‐power field.

* 24 MGH cases stained;

** one case showed focal subnuclear vacuoles;

*** one case showed staining in squamous differentiation, three cases showed focal non‐specific staining;

**** 22 EMCA cases stained.

**Figure 4:**
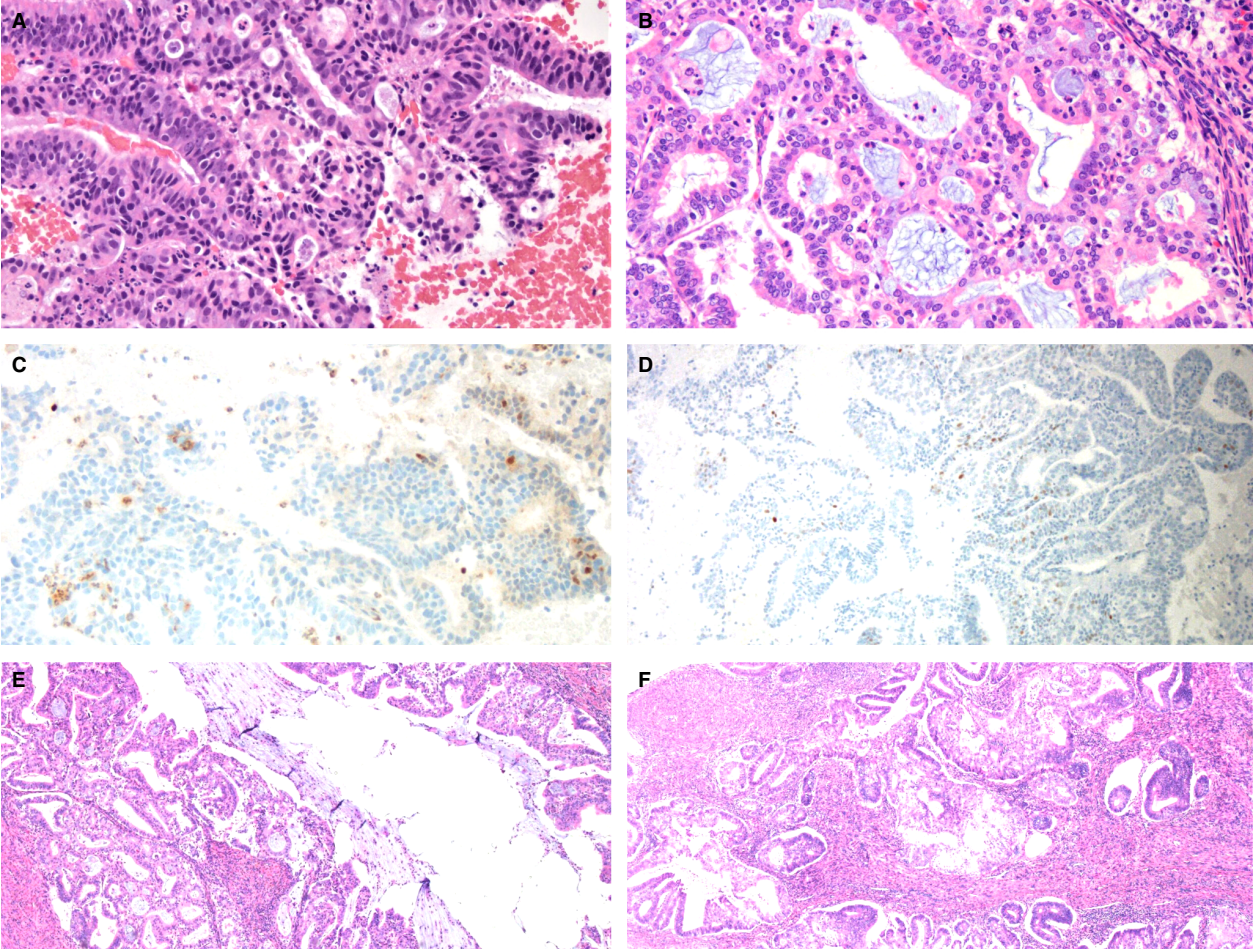
Morphological and immunohistochemical features of atypical microglandular proliferation with endometrioid carcinoma on follow‐up. Some atypical microglandular proliferations showed morphological features considered insufficient for more specific classification, including small tightly packed glands with mild cytological atypia and prominent neutrophilic inflammation [**A**,**B,** haematoxylin and eosin (H&E)]. This case showed loss of phosphatase and tensin homologue (PTEN) expression [**C**, PTEN immunohistochemistry (IHC)] and focal non‐specific p63 staining (**D**, p63 IHC). The subsequent hysterectomy (**E**,**F**, H&E) confirmed the diagnosis of endometrial endometrioid carcinoma.

**Figure 5:**
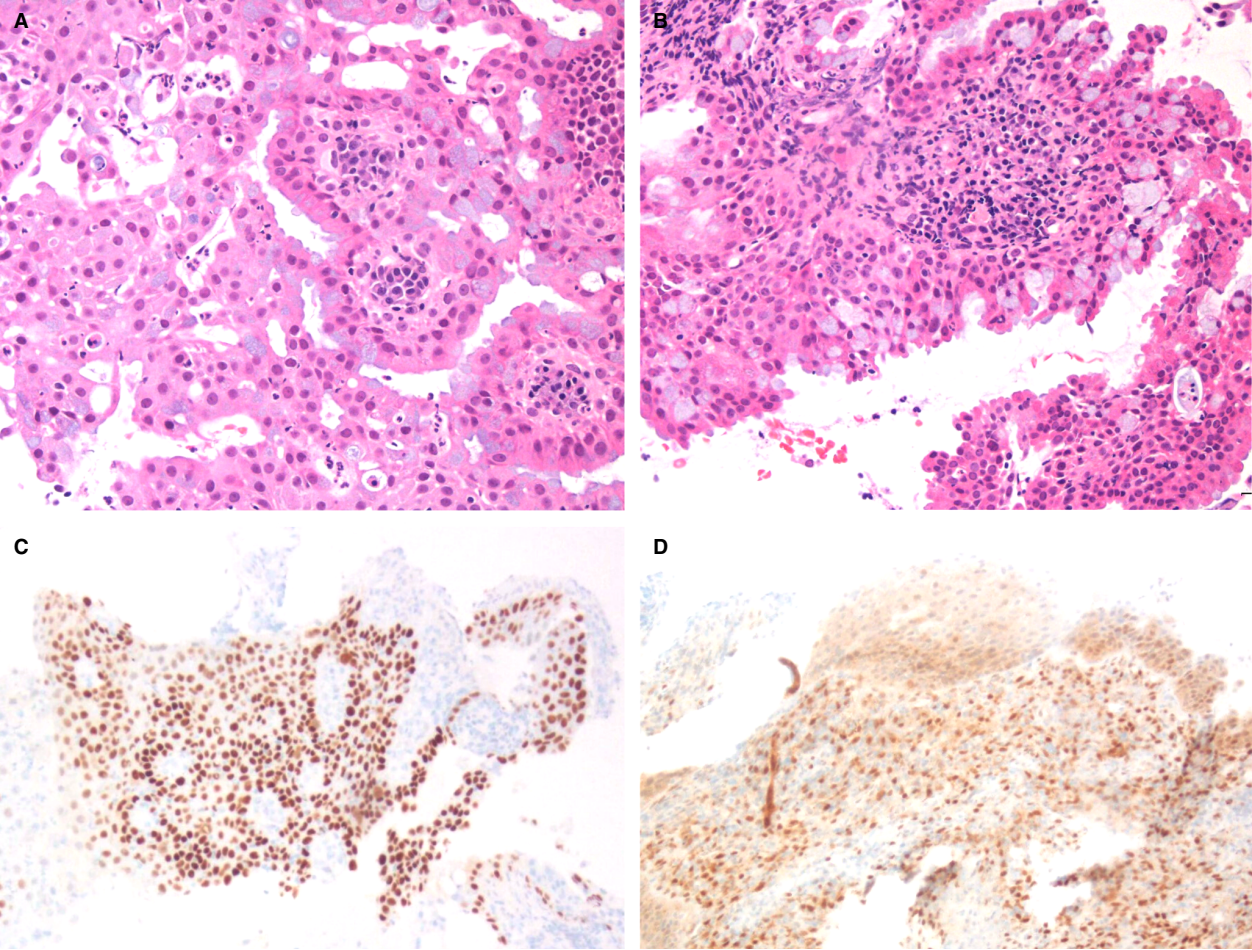
Morphological and immunohistochemical features of atypical microglandular proliferation with microglandular hyperplasia on follow‐up. The biopsy shown in panel (**A**) was diagnosed as an atypical microglandular proliferation given the small, tightly packed glandular spaces, squamous and mucinous features, and acute inflammation [haematoxylin and eosin (H&E)]. A follow‐up biopsy more clearly showed features of microglandular hyperplasia (**B**, H&E). p63 highlighted squamous cells and reserve cells [**C**, p63 immunohistochemistry (IHC)], and phosphatase and tensin homologue (PTEN) expression was retained (**D**, PTEN IHC).

Nine cases in our cohort were diagnosed as ‘atypical microglandular proliferations’, as the signing pathologists could not confidently classify them based on morphological examination and consensus review. Detailed information about these cases is displayed in Table 2. Subsequent diagnostic specimens were available for three patients, two with EMCA and one with MGH. The two cases with EMCA on follow‐up were essentially negative for p63 with loss of PTEN expression in one follow‐up case (Figure 4A–F), and loss of PTEN expression with focal retention in the second follow‐up case. The case with MGH on follow‐up demonstrated subcolumnar p63 staining and intact PTEN expression (Figure 5A–D). Of the remaining six atypical microglandular proliferations, two showed staining patterns suggestive of EMCA, three showed subcolumnar p63 staining and intact PTEN expression suggestive of MGH, and one showed focal non‐specific p63 positivity with intact PTEN expression.

**Table 2:** Clinical, morphological and immunohistochemical features of atypical microglandular proliferations

Patient history					Morphological description								Immunohistochemical stain		Follow‐up diagnosis
Case	Age	Hormone therapy use	Postmenopausal?	Site/procedure	Gland architecture	Luminal secretions/mucin	Subnuclear vacuoles	Foamy histiocytes	Inflammation	Luminal squamous metaplasia	Cytological atypia	Mitotic figures per 10 HPG	p63	PTEN	
1	93	NA	Yes	Endometrium/curettage	Small, tightly packed	Present	Absent	Present	Moderate	Present	Absent	0	Positive/subcolumnar	Intact	NA
2	76	NA	Yes	Endometrium/biopsy	Small, tightly packed	Present	Absent	Present	Absent	Absent	Absent	1	Positive/subcolumnar	Intact	NA
3	72	None	Yes	Endometrium/biopsyHysterectomy (follow‐up)	Medium and tubular, tightly packed, occasionally fused	Present	Absent	Present	Moderate	Absent	Present	3	Negative	Lost	EMCA FIGO Grade 1
4	58	None	Yes	Endometrium/biopsy	Small, tightly packed	Present	Absent	Absent	Moderate	Absent	Absent	0	Positive/focal subcolumnar	Intact	NA
5	70	None	Yes	Endometrium/biopsy and curettageHysterectomy (follow‐up)	Variable in size, tubular, tightly packed	Present	Absent	Present	Moderate	Absent	Present	6	Negative	Lost (focally intact)	EMCA FIGO Grade 1
6	45	None	No	Endometrium, Cervix/ biopsy and curettage	Small, tightly packed	Present	Absent	Present	Moderate	Present	Absent	0	Positive/subcolumnar	Intact	MGH
7	47	OCP	No	Endometrium/biopsy	Small to medium, tightly packed	Absent	Absent	Absent	Absent	Absent	Present	0	Negative	NA	NA
8	66	None	Yes	Endometrium/biopsy	Small, tightly packed	Present	Absent	Absent	Severe	Absent	Present	2	Positive (focal)	Intact	NA
9	66	None	Yes	Endometrium/curettage	Small to medium, tightly packed	Present	Absent	Absent	Moderate	Absent	Present	2	Negative	Lost	NA

EMCA, endometrial endometrioid carcinoma; MGH, microglandular hyperplasia; PTEN, phosphatase and tensin homologue; NA, not available; OCP, oral contraceptive pill.

## Discussion

As has been established in the literature, there is significant morphological overlap between benign endocervical MGH and low‐grade EMCA with microglandular and/or mucinous features.^1^, ^6^, ^12^, ^14^, ^19^ Similar to previous studies, cytological atypia, mitotic activity, foamy histiocytes and medium to large complex glands were seen in the majority of EMCA cases in our cohort, whereas small tightly packed glands and subnuclear vacuoles were more commonly seen in MGH cases. Only two cases of MGH in our cohort demonstrated rare mitotic activity, emphasising another source of diagnostic uncertainty.^4^, ^19^

Again, immunohistochemical stains are often described as having limited utility in this diagnostic setting. Vimentin expression is more common in EMCA; however, vimentin staining may be patchy in EMCA and can also rarely be seen in MGH.^12^, ^13^, ^20^ Expression of oestrogen receptor and progesterone receptor is variable in both entities.^4^, ^12^, ^13^, ^20^ Stewart *et al*. reported that a subset of EMCA show PAX2 loss, although the diffuse reactivity in MGH lesions was consistently weak.^13^ Chekmareva *et al*. demonstrated that CD10 and CD34 can aid the distinction between endometrial (CD10‐positive) and endocervical (CD34‐positive) stroma.^20^ Pavlakis *et al*. found that p63 reactivity was more common in MGH than EMCA, although the percentage of p63‐positive cells, rather than the pattern of staining, was reported.^12^ Importantly, all MGH cases in our cohort displayed p63‐positive subcolumnar reserve cells. Only four EMCA cases showed focal p63 staining, primarily at the surface of the tumour and in areas of squamous differentiation.

To further elucidate the nature of these proliferations, PTEN staining was performed. The majority of EMCA cases demonstrated loss of PTEN expression by immunohistochemistry, while intact reactivity for PTEN was seen in all but one case of MGH. Furthermore, eight of nine cases initially diagnosed as ‘atypical microglandular proliferations’ could be diagnosed as either EMCA or MGH based on p63 and PTEN staining patterns. Three such cases had subsequent hysterectomies, confirming that classification based on p63 and PTEN staining patterns was accurate. However, further investigation of staining patterns in atypical microglandular proliferations with known follow‐up is required to confirm the specificity of these findings. Of note, the proportion of our EMCA cases demonstrating loss of PTEN expression by immunohistochemistry is higher than that generally reported in the literature. However, it has been reported that PTEN is abnormal in 91.9% of FIGO grade 1 EMCA when both IHC and mutational data are considered.^17^

Strengths of this study include the relatively large cohort of EMCA with microglandular and/or mucinous features and the use of whole tissue sections for immunohistochemical work‐up. Limitations of this study include the lack of subsequent specimens for six of the biopsies diagnosed as ‘atypical microglandular proliferation’. However, based on the findings from established MGH and EMCA cases and atypical microglandular proliferations with available follow‐up, the p63 and PTEN immunohistochemical staining patterns appear to be helpful. Of note, MGH may coexist with EMCA in some cases, so close attention should be paid to the tissue fragments of interest on biopsy or curettage specimens.

In summary, p63 immunohistochemistry is beneficial for highlighting subcolumnar reserve cells and confirming a diagnosis of benign endocervical microglandular hyperplasia. In challenging cases (such as MGH cases with a less obvious reserve cell layer or EMCA cases with non‐specific p63 staining), PTEN immunohistochemistry can provide additional useful information.

## Conflicts of interest

None.

## Supporting information


**Table S1.** The distribution of histologic diagnoses in association with specimen type.

## Data Availability

The data that support the findings of this study are available from the corresponding author upon reasonable request.
