# Supplementary material for: Utility of p63 and PTEN staining in distinguishing cervical microglandular hyperplasia from endometrial endometrioid carcinoma with microglandular/mucinous features
Source: Histopathology. 2022 May 5;80(7):1102–11. doi: 10.1111/his.14655 (PMC9322015; doi:10.1111/his.14655)
Supplement: Supplementary file 1 — Table S1. The distribution of histologic diagnoses in association with specimen type. [file HIS-80-1102-s001.docx]

Supplemental Table. The distribution of histologic diagnoses in association with specimen type.

|  | Total hysterectomy | Endometrial biopsy | Endometrial curettage | Cervical biopsy | Endocervical curettage | LEEP* |
| --- | --- | --- | --- | --- | --- | --- |
| Endometrial Endometrioid carcinoma (EMCA) | 19** | 4 | 4 | 0 | 0 | 0 |
| Endocervical Microglandular hyperplasia (MGH) | 0 | 1 | 2 | 7 | 16 | 1 |
| Atypical Microglandular proliferation | 0 | 7 | 2 | 0 | 0 | 0 |

^*^Loop electrosurgical excision procedure

**Includes 3 follow-up hysterectomy specimens
